# Explaining adverse cholesterol levels and distinct gender patterns in an Indonesian population compared with the U.S.

**DOI:** 10.1016/j.ehb.2024.101403

**Published:** 2024-08

**Authors:** Ralph Lawton, Elizabeth Frankenberg, Teresa Seeman, Arun Karlamangla, Cecep Sumantri, Duncan Thomas

**Affiliations:** a Harvard University, Cambridge, MA, United States; b University of North Carolina at Chapel Hill, Chapel Hill, NC, United States; c David Geffen School of Medicine at UCLA, Los Angeles, CA, United States; d SurveyMeter, Yogyakarta, Indonesia; e Duke University, Durham, NC, United States

**Keywords:** Cardiovascular risks, Biomarkers, Cholesterol, Indonesia, United States

## Abstract

Cardiovascular disease is among the most common causes of death around the world. As rising incomes in low and middle-income countries are accompanied by increased obesity, the burden of disease shifts towards non-communicable diseases, and lower-income settings make up a growing share of cardiovascular disease deaths. Comparative investigation of the roles of body composition, behavioral and socioeconomic factors across countries can shed light on both the biological and social drivers of cardiovascular disease more broadly. Comparing rigorously-validated measures of HDL and non-HDL cholesterol among adults in the United States and in Aceh, Indonesia, we show that Indonesians present with adverse cholesterol biomarkers relative to Americans, despite being younger and having lower body mass index. Adjusting for age, the gaps increase. Body composition, behaviors, demographic and socioeconomic characteristics that affect cholesterol do not explain between-country HDL differences, but do explain non-HDL differences, after accounting for medication use. On average, gender differences are inconsistent across the two countries and persist after controlling observed characteristics. Leveraging the richness of the Indonesian data to draw comparisons of males and females within the same household, the gender gaps among Indonesians are not explained for HDL cholesterol but attenuated substantially for non-HDL cholesterol. This finding suggests that unmeasured household resources play an important role in determining non-HDL cholesterol. More generally, they appear to be affected by social and biological forces in complex ways that differ across countries and potentially operate differently for HDL and non-HDL biomarkers. These results point to the value of rigorous comparative studies to advance understanding of cardiovascular risks across the globe.

## Highlights

• Relative to Americans, Indonesians have lower HDL and higher non-HDL cholesterol.
• This suggests Indonesians have elevated CVD risks relative to Americans.
• Behaviors, anthropometry, and medication use explain cross-country gaps in non-HDL.
• But, the same characteristics do not explain cross-country gaps in HDL.
• Indonesian women have higher non-HDL than men, the reverse of the U.S. differences.

## 1 Introduction

Global deaths from cardiovascular disease are rising at alarming rates, driven in part by elevated mortality risks in low and middle-income countries as a result of the epidemic of obesity (Popkin, 2001, Roth et al., 2015). In recent years, increasing risk factors for and mortality from cardiovascular disease among Asian and South Asian populations has been a source of substantial concern in the United States (U.S.) population and globally (Jose et al., 2014, Taddei et al., 2020, Volgman et al., 2018).

However, while substantial cross-country variation in cardiovascular disease has been documented, the mechanisms underlying these differences are not well understood, and there is a paucity of evidence on disparities by gender as the burden of disease shifts from males to females in many countries (Vogel et al., 2021). As low and middle-income countries undergo social and economic transformation, they provide unique environments to examine the biological, behavioral, and socioeconomic mechanisms involved in cardiovascular disease and health disparities. The relationships between anthropometry and cardiovascular disease have been studied across contexts, which has revealed differences in these relationships between males and females (Goryakin et al., 2017, Rashad, 2008). Examining populations that do not follow the ‘typical’ patterns that characterize higher-income countries may be scientifically very valuable to advance understanding of cardiovascular disease (CVD) in the U.S. and abroad (Frankenberg et al., 2016, Godoy et al., 2007, Schooling and Leung, 2010, Zeljko et al., 2013).

We compare levels of high-density lipoprotein (HDL) and non-HDL cholesterol, two biomarkers fundamentally associated with increased risk of cardiovascular disease, in population-representative samples of adults in Aceh, Indonesia and adults in the U.S. Several characteristics make the Indonesian population an interesting comparison with the U.S. Some of the most important factors that affect lipid levels and cardiovascular disease risk include age and aspects of body composition such as BMI and waist circumference, which differ across our contexts (Haskell et al., 1980, Le-Ha et al., 2013, Lew et al., 2017, Wilson et al., 1998). Although the Indonesian population is younger and has a more favorable body composition than the American population, Indonesians have cholesterol profiles that suggest higher risks of CVD relative to Americans.

Low HDL cholesterol and high non-HDL cholesterol are indicative of elevated CVD risks: Indonesians have worse profiles on both biomarkers, on average, relative to Americans. The differences between the two populations are substantively large. For example, HDL cholesterol levels are about 6 mg/dL lower among Indonesians in our sample relative to Americans, a magnitude linked in prior studies to approximately 14% increased risk of death due to CVD. Relative to Americans, an additional 13% of women and 25% of men in Indonesia fall below the widely-used clinical cut-off for a healthy level of HDL cholesterol of 40 mg/dL (Cui et al., 2001). The results for Indonesia contrast with findings from other Asian contexts, which typically find lower HDL but also lower non-HDL than in the U.S. Further, patterns by gender across countries are completely different. Indonesians have an inverted non-HDL cholesterol gender gap relative to Americans in the sense that U.S. females are at lower risk relative to males whereas Indonesian females are at higher risk relative to males.^1^

The reasons for these differences in patterns across the populations are a puzzle. We investigate several potential explanations. Specifically, we examine the relationships between age, gender, body composition, socioeconomic factors, and cardiovascular disease risk in Indonesia, and draw comparisons with a parallel sample in the U.S. To further interrogate the ‘inverted’ gender disparities in non-HDL cholesterol, we draw contrasts between males and females living in the same household at the time of measurement. These estimates abstract from the effects of household resources and structure, as well as environmental factors and other unobserved characteristics and behaviors that are shared within households and typically very difficult to measure. We show these unobserved characteristics likely underlie the distinctive patterns by gender across the two countries.

Whereas the existing global health literature documents differences in risk and disease profiles across countries and regions throughout the world and points to the importance of understanding the reasons for these differences, this study contributes to the literature by conducting a systematic and in-depth comparison of the roles of biology, behavior and socioeconomic factors in explaining differences in validated biological markers of cardiovascular risks across vastly different contexts (Roth et al., 2015, Roth et al., 2020, Taddei et al., 2020). The literature documents that the relationships between socioeconomic status and self-rated health or self-reported diagnoses vary from country to country and, in many contexts, between males and females. This variation has often been interpreted as reflecting that gender-related social forces and resource-related access to health care play important roles in reported differences (Assari, 2014, Moghani Lankarani et al., 2017, Witoelar et al., 2009). This makes measurement with biological health markers essential, as self-reported disease risk can be endogenously related to social strata, access to resources and use of health care.

Accordingly, we leverage detailed population-representative data that we collected from 5,577 adults in Aceh, Indonesia, using validated point-of-care instruments, and draw comparisons with 4,937 American adults assessed in the National Health and Nutrition Examination Survey (NHANES). In order to ensure the quality of the data we collected, we conducted a rigorous validation of the point-of-care instrument used to measure cholesterol in the field. Prior to data collection, we established that high humidity and temperature result in the measures being systematically downward biased. We therefore developed a mobile laboratory that was climate-controlled and drew samples and conducted the assay in the mobile lab. Those measures of cholesterol match measures from paired samples tested in a commercial laboratory. This is an important methodological contribution of our research since very few population-based studies that have collected biomarkers in field settings have validated the procedures (Thomas et al., 2018).

Our results document the importance of body composition and behavioral factors and, despite significant economic change in Indonesia over the past several decades, find little role for socioeconomic factors in explaining the differences between cholesterol profiles of Americans and Indonesians. We highlight the importance of distinguishing HDL from non-HDL cholesterol and establish that differences in HDL cholesterol of Americans and Indonesians cannot be attributed to a broad array of observed differences but, rather, indicate that there are likely differences in the underlying health production functions. We also find large unexplained differences in gender gaps across the two countries, which we suggest in Indonesia can be largely attributed to shared household-level characteristics.

These comparisons are important because, as lower resource economies develop, and the nutrition transition continues its march around the globe, understanding the ramifications of the attendant changes in society on cardiovascular disease is essential. Indonesia has recently experienced rapid recent economic growth and a parallel epidemiological transition in its disease burden (one in three deaths are now attributable to cardiovascular disease), including transitions in disease burden across socioeconomic strata (Aizawa and Helble, 2017, Witoelar et al., 2009). As a result, Indonesia makes a high-value case study. Detailed comparisons between higher and lower-resource countries provide the opportunity to improve understanding of the unique linkages between gender, biology, and social mechanisms that drive cardiovascular disease risk in Indonesia, the U.S., and around the globe.

## 2 Conceptual framework and empirical methods

### 2.1 HDL and non-HDL cholesterol

We focus on levels of HDL and non-HDL cholesterol, as they are core biomarkers for cardiovascular risk. Atherosclerosis, the buildup and hardening of plaques in the arteries which can eventually occlude vessels, break off, or rupture, is the primary underlying cause of heart attack and stroke. Simply, plaques are formed in several steps: 1) infiltration of subsets of non-HDL cholesterol into blood vessel walls, where they are oxidized 2) macrophages consume cholesterol molecules, leading to foam cell formation, 3) inflammation from foam cells results in elevated oxidative stress which leads to additional LDL oxidation, foam cell formation, and proliferation of this process, 4) foam cell recruit smooth muscle cells to form a fibrous barrier between the plaque and the bloodstream. This process can then lead to heart attacks or strokes when occlusion of blood vessels or rupture of blood vessels occur – typically when expanding plaques occlude blood vessels, or ruptured fibrous barriers lead to sudden clotting when platelets in the blood contact the plaque directly (either occluding blood vessels or creating clots that occlude smaller vessels downstream). HDL cholesterol plays an important role in this process by reverse cholesterol transport – helping to clear non-HDL cholesterols that may be part of plaque formation (Linton et al., 2000).

Due to their essential roles in the pathophysiology of cardiovascular disease, blood HDL cholesterol and non-HDL cholesterol levels are used as proxies for risk. They have both been linked extensively to clinical outcomes and are common first-line targets of primary care. Low levels of HDL cholesterol are associated with increased risk of cardiovascular disease incidence, and mortality including coronary heart disease, coronary artery disease, and stroke (Barter and Rye, 1996, Castelli et al., 1986, Cooney et al., 2009, Gordon and Rifkind, 1989, Weverling-Rijnsburger et al., 2003). High non-HDL cholesterol levels are linked to coronary artery disease, and at some ages increased risk of cardiovascular and all-cause mortality (Anderson et al., 1987, Weverling-Rijnsburger et al., 2003). Large-sample genetic evidence reinforces the findings from clinical and population studies, linking genetic predictions of HDL and non-HDL cholesterol to aortic aneurysm risk (Klarin et al., 2018).

A paucity of population-representative evidence on the links between cholesterol levels and cardiovascular disease risks in the Indonesian population makes it difficult to draw firm conclusions about the clinical relevance of the differences in cholesterol levels that we document. However, evidence from similar contexts suggests the biomarkers are powerful predictors of cardiovascular disease and the differences we document are important. From a biological perspective, there is little reason to believe that these biomarkers would play substantially different physiologic roles in Indonesia compared with the U.S. Among South Asians, adverse communicable disease environments and elevated background inflammation may play a role in increased risk for a given cholesterol level (Stefil et al., 2023). Epidemiologic study of other Asian populations suggests relationships between these biomarkers and mortality, heart disease, and stroke that are similar to those observed in American populations (Asia Pacific Cohort Studies Collaboration, 2003). Further, results from randomized trials of statin efficacy, which primarily lowers non-HDL cholesterol, document similar efficacy across racial groups including Asian populations (Albert et al., 2011, Kushiro et al., 2009). Taken together, while the evidence on CVD risks at a given cholesterol level across countries is not conclusive, parallel international shifts in the distribution of cholesterol risk and recorded CVD, knowledge of inflammatory environments, and clinical trials of statins suggest these biomarkers are important in contexts similar to Indonesia.

### 2.2 Etiology of HDL and non-HDL cholesterol

Non-HDL cholesterol levels can be shaped by a variety of factors. After puberty, non-HDL levels rise significantly with age, before plateauing and decreasing slightly later in life (Kreisberg and Kasim, 1987). In most populations, non-HDL increases more rapidly with age in males than females. As a result, non-HDL levels among males are typically lower than among females (Kreisberg and Kasim, 1987). Estrogen likely plays an important role in improved cholesterol levels in women compared with men. Adiposity is linked to adverse non-HDL levels, potentially through a variety of mechanisms including the effects of adiposity on decreasing HDL levels, increased absorption of cholesterol, and downregulation of enzymes that clear non-HDL cholesterol in adipose tissue (Auley, 2020, Klop et al., 2013). A variety of lifestyle factors also contribute to non-HDL cholesterol levels, including diet, physical activity, and smoking (Caldwell et al., 2019). Of note, smoking’s impact on non-HDL levels may potentially be mediated by adverse effects of smoking on HDL (Campbell et al., 2008, Muscat et al., 1991). Non-HDL cholesterol is also the primary target of statin drugs, first-line agents for preventing atherosclerosis that dramatically lower non-HDL cholesterol levels. In short, statin drugs reduce de novo synthesis of cholesterol, leading to increased cellular absorption of non-HDL cholesterol out of the bloodstream.

HDL cholesterol has many of the same risk factors as non-HDL cholesterol, in part because some of the mechanisms that adversely affect non-HDL cholesterol do so by downregulating HDL cholesterol. Large-sample genetic studies also find similar proportions of the variation of HDL and non-HDL cholesterol can be explained with genetic markers (11% and 9%, respectively) (Klarin et al., 2018). There are, however, several notable differences. First, while there are strong age gradients in non-HDL cholesterol, age gradients for HDL differ for males and females: they are typically relatively flat for females, while for males they decrease rapidly during puberty and early adulthood and then remain relatively flat from that point. Clinical studies that suppress testosterone suggest a potentially causal role of testosterone in lowering men’s HDL levels (Bagatell et al., 1992, Semmens et al., 1983). Second, while smoking may affect non-HDL cholesterol levels, its primary impact is on HDL cholesterol (Maeda, Noguchi, and Fukui, 2003). Third, while statin drugs can have dramatic impacts on non-HDL cholesterol levels, their effects on HDL cholesterol are very small, potentially increasing levels slightly (Barter et al., 2010). This difference emphasizes that while the regulation of HDL and non-HDL cholesterol are related, they are governed by different processes.

### 2.3 Conceptual and empirical models

Although the Indonesian population is younger, has lower body mass and less abdominal fat, factors that have been shown to be linked to less adverse cholesterol profiles, Indonesians have worse HDL cholesterol and non-HDL cholesterol than Americans. Further, while, relative to females, males have adverse cholesterol levels in the U.S., in Indonesia females have slightly worse non-HDL cholesterol than males. These facts motivate an exploration of the potential mechanisms at play, which to some extent challenge typically assumed facts about the production function of cardiovascular risk, and illuminate the ways in which it may differ across contexts. To formalize our approach to understanding what may drive these differences, we leverage the following conceptual and empirical models of cardiovascular health.

Assume an individual allocates resources, including own time, at each point in the life course to maximize lifetime well-being, including health, given resource and information constraints the individual faces at that point in time. Well-being includes health which is constrained, at each point in time, by a dynamic health production function that depends on inputs over the life course such as food intake and diet quality, health-related behaviors including quantity and quality of health care use, exercise and smoking.

In the model, health outcomes may also depend on individual-specific characteristics and the local area environment. These characteristics may have a direct effect on health, they may affect the shape of the health production function and/or they may interact with inputs or each other. Individual-specific characteristics include, for example, age, gender and education, resources over the life course, genetic factors and family background. Local area factors include the quality of the environment, availability of health care services, the prices of health care and prices of all other goods. These individual and local-area factors potentially play a role reaching back to in utero exposures. Future characteristics may also be relevant through the role of future expectations affecting prior behavior.

Solving this dynamic optimization problem yields a demand function for a health outcome at a point in time which depends on exogenous factors that affect health through the health production function and/or the time, budget and information constraints. A primary goal of this research is to advance understanding of the relationships between cardiovascular risks, as indicated by measures of cholesterol, and individual-level characteristics by drawing comparisons between populations in the U.S. and Indonesia. There are large differences in several key inputs into the health production function between the two populations: for example, in our Indonesian sample, average BMI is about 25 kg/m^2^ whereas in the U.S. sample it is 30 kg/m^2^. Failing to adjust for these observed differences across the populations has the potential to hide important explanations for differences in cholesterol levels. Therefore, we estimate conditional demand for health outcome functions that adjust for these differences (Pollak, 1969):

[1] $$C_{i}=\theta^{P}(N_{i},\;\;Z_{i,}\;\;\xi_{i})$$

where $C_{i}$ is measured HDL and non-HDL cholesterol of respondent *i* in population P at the time of the survey, $N_{i}$ are the conditioning inputs such as BMI, smoking behavior, medication use and physical activity, and $Z_{i,}$ are individual and area-level characteristics. The health production function is represented by $\theta^{P}$ which we do not restrict to be the same across populations, P. Unobserved heterogeneity that affects health outcomes of individual *i* in population P is captured in $\xi_{i}$.

Linearizing [1] and collecting the observed characteristics in the production functions, $N_{i}$ and $Z_{i,}$in a vector of covariates,$X_{i}$we estimate the linear regression for each population, P,

[2] $$C_{i}=\alpha_{0}^{P}+X_{i}\;\;\alpha_{1}^{P}+\;\varepsilon_{i}^{P}$$

allowing the health production functions and conditional demand functions to differ across populations. Unobserved factors in the empirical models are captured by $\varepsilon_{i}$.

To facilitate comparisons across the U.S. and Indonesian populations, we also report estimates with samples from both populations, allowing the coefficient estimates to differ across the two populations:

[3] $$C_{i}=\beta_{0}+\;X_{i}\;\beta_{1}+I_{i}\beta_{2}+X_{i}I_{i}\beta_{3}+\;\varepsilon_{i}$$

where the indicator variable $I_{i}$ identifies respondents from Indonesia (taking the value one for those respondents and zero for U.S. respondents). In [3], each element of the vector of estimates, ${\hat{\beta }}_{3}$, is equivalent to the corresponding difference in the estimates ${\hat{\alpha }}_{1}^{I}-{\hat{\alpha }}_{1}^{US}$in models [2], where the superscripts I and US refer to the Indonesian and U.S. populations.

After presenting results from estimating [2] and [3], we explore the extent to which the differences in cholesterol levels can be attributed to differences in the observed characteristics, $X_{i}$, across the populations. Since both HDL and non-HDL cholesterol vary with age, and the Indonesian population is substantially younger than the U.S. population, we begin by adjusting only for age. We add the other controls and adopt a Blinder-Oaxaca decomposition to separate the differences across the population into the part that can be attributed to observed differences in covariates and the part that cannot be thus explained (Blinder, 1973, Oaxaca, 1973).

The literature has documented that the relationships [2] differ by gender within many populations, including the U.S. Since we find that to also be true in Indonesia, all empirical models are reported separately for males and females. We further explore these gender differences by exploiting the fact that in the Indonesian survey, we measure cholesterol for all age-eligible participants in each sample household. Drawing comparisons between males and females living in the same household at the time of the survey has the advantage of sweeping out the effects of shared observed and unobserved household and local-area factors that affect cholesterol in a linear and additive way. These include, for example, factors such as diet and nutrition, local area prices and access to health services, which are not included in the models because they are not observed in both datasets. Diet is of particular interest in this context since it has the ability to directly shape cholesterol levels, and has substantial socioeconomics gradients, particularly in lower-resource settings (Rahkovsky and Gregory, 2013, Worku et al., 2017).

Specifically, we estimate models that related cholesterol measures of individual *i* in household *h*, $C_{ih}$. to the subset of covariates in [2], that vary within a household,$X_{ih}$, controlling gender of the respondent, $M_{ih}$, which is also is interacted with the covariates in model [2]. The and including a household-specific fixed effect, $\mu_{h}$:

[4] $$C_{ih}=\delta_{0}+\;X_{ih}\;\delta_{1}+M_{ih}\delta_{2}+X_{i}M_{ih}\delta_{3}+\;\mu_{h}+\varsigma_{ih}$$

where unobserved heterogeneity is $\varsigma_{ih}$. While the household fixed effect absorbs the influence of shared characteristics that affect cholesterol, the models allow the relationships between the individual-level characteristics that affect cholesterol to vary with gender and also allow a gender differential in the association with observed household-level characteristics. For example, the relationship between household resources and cholesterol that is common for all household members is captured in the fixed effect; estimates of the coefficient on the interaction terms, $\delta_{3}$, reflect the differences in these associations between males and females. These differences may reflect biological differences, an efficient allocation of resources within the household and they may reflect differential bargaining power. These analyses are restricted to the Indonesian survey since the NHANES public-use files do not include household-specific identifiers.

## 3 Data

### 3.1 Study populations

We draw on population-representative data from the U.S. and Indonesia. The Indonesian data are a subsample of the Study of Tsunami Aftermath and Recovery (STAR), a longitudinal survey of individuals who were living along the coast of Aceh and North Sumatra, Indonesia, at the time of the Indian Ocean earthquake and tsunami on December 26, 2004. STAR follows survivors of the pre-tsunami baseline collected in February/March 2004 by Statistics Indonesia as part of SUSENAS, an annual socioeconomics survey.

In order to collect detailed information about biological health risks, we selected a random sub-sample of respondents in 2017–2018 and measured non-fasted total and HDL cholesterol. The sample is a 25% random sub-sample of 411 enumeration areas in Aceh included in the 2004 baseline with, overweighting of areas that sustained the most damage from the tsunami. All baseline survivors and their children born after the tsunami who were age 8 and older at the time of the 2017–18 follow-up were eligible for the biological risks sub-study. This includes people who moved away from the pre-tsunami community of residence. Of these individuals, 93.5% agreed to participate. A trained phlebotomist collected venous blood from each respondent in a small mobile lab that was parked outside the respondent’s home.

While our data is drawn from the province of Aceh, the overall patterns of cholesterol levels and gender epidemiology, in particular the inverted gender gaps in non-HDL cholesterol, match population-level data from other studies in Asia and Indonesia. The distinctive epidemiology in this population provides insightful comparisons with the U.S., and we use rich household-linked data in the Indonesian sample to more deeply study the intersections of socioeconomic status and gender with cholesterol levels. However, it is important to recognize our results may not generalize to all of Indonesia.

To draw comparisons with the U.S., this study uses the 2017–2018 wave of the National Health and Nutrition Examination Survey (NHANES). The NHANES is a nationally representative cross-sectional sample of American adults and children, with in-depth data on health and nutritional status. Cholesterol measures were collected on individuals 6 and older.

Sample weights for both populations are used so that the analyses are representative of the target populations in Aceh and the U.S. The STAR weights take into account the sampling probabilities of the selected pre-tsunami enumeration areas so that the weighted statistics represent the populations in the districts included in STAR. NCHS-provided sample weights for the NHANES Mobile Examination Clinic sample provided by the NCHS are used for the NHANES analyses. Results are reported for adult respondents age 20 y or older at the time of measurement in both populations.

### 3.2 Validation of cholesterol measurement in the field in Indonesia

We draw comparisons between Indonesia and the U.S. for two biomarkers, HDL cholesterol and non-HDL cholesterol, considered core predictors of cardiovascular disease risk. Both are markers of population health and of interest to health and social scientists. They are routinely collected in the course of most primary care practices as well as measured in population-representative broad-purpose studies such as the Health and Retirement Survey (HRS), Demographic and Health Surveys (DHS), Indonesian Family Life Survey (IFLS), Health and Aging in Africa (HAALSI), the Cebu Longitudinal Health and Nutrition Survey (CLHNS), and others (Gaziano et al., 2017, Mishra et al., 2009, Strauss et al., 2009).

When comparing levels of biomarkers across studies, it is important to establish that differences are not driven by systematic differences in measurement. In STAR, cholesterol is measured with the CardioChek POCT. It is widely used in clinical settings and has been validated in controlled clinical settings although concerns have been raised with its performance in clinics in some lower-resource settings (Ferreira et al., 2015, Gao et al., 2016, Kuzawa et al., 2019, Park et al., 2016). The CardioChek has also been used in the field in several large-scale and important population-based studies including CLHNS, HAALSI and IFLS. Unfortunately, those studies have not published validation studies, although there is evidence that field conditions can substantially bias biomarker measures (Thomas et al., 2018).

We therefore designed a protocol to rigorously evaluate the performance of the CardioChek in our field setting prior to conducting fieldwork. Specifically, we measured performance in three settings: (1) a well-controlled clinical or “ideal” setting, (2) “field” settings that were community centers with little or no climate control located near respondents’ homes and replicated measurement in the respondent’s home (3) in a “mobile lab” that we constructed by placing the sample collection materials and equipment in a minivan that was carefully climate controlled with a portable air conditioning system and having the samples drawn in the “mobile lab”. The mobile lab was parked outside or close to each respondent’s home. For each setting, we conducted a paired-sample analysis, with samples from the same respondent being analyzed with the CardioChek and at a gold-standard commercial laboratory, Prodia, located in the province capital of Banda Aceh. (The “ideal” setting was located adjacent to the Prodia lab.)

**Fig. 1:**
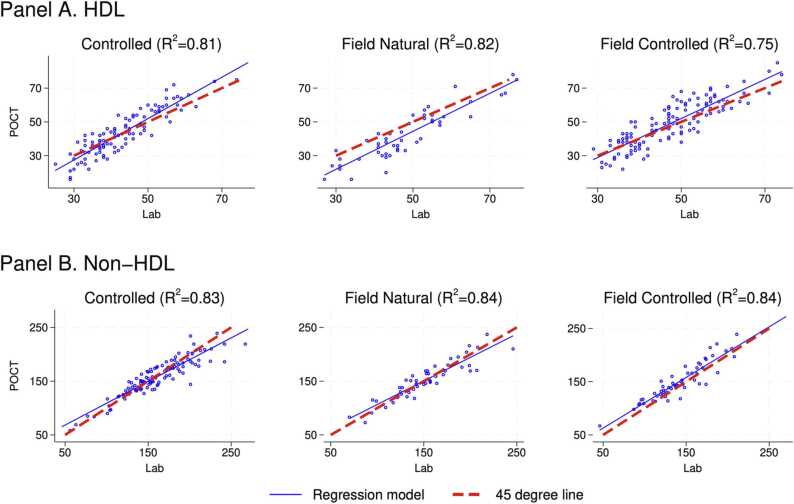
CardioChek Cholesterol Performance Across Conditions.

POCT results from the “ideal” setting validated well. While measurement was noisier than the gold-standard clinical laboratory, the R^2^ of the paired samples was high for both HDL cholesterol and non-HDL cholesterol (0.81 and 0.83, respectively) and was unbiased, showing initial promise for CardioChek as an efficient way to measure cholesterol in the field for population studies (Fig. 1, Panels A and B). However, performance in the “field” setting was significantly poorer, in particular for HDL cholesterol which was significantly downward biased. For example, on average, HDL cholesterol was 6.2 mg/dL lower than the gold standard value (48 mg/dL) (Appendix Table 1). This had important implications for the classification of individuals into high or low-risk categories (Appendix Table 2). In the “field” conditions, the CardioChek incorrectly classified 44% of individuals in the ‘normal’ HDL range as having clinically significant low HDL (Appendix Table 2). However, while HDL was significantly downward-biased, the performance of non-HDL assessment under uncontrolled field settings was much better, and was not significantly different from the gold-standard values.

Performance of CardioChek was far superior in the “mobile lab.” It matched the gold standard in terms of HDL levels (Appendix Table 1) and classification of low HDL levels (Appendix Table 2). While the non-HDL cholesterol POCT performance in the controlled field setting had an intercept at the mean significantly different from 0, relative to the mean of 149.8, the differences were relatively small. Further, the slope was closer to 1 than in the ideal conditions, and the POCT was able to accurately classify high and normal levels of non-HDL cholesterol (Fig. 1 and Appendix Table 2).

We therefore assessed HDL and non-HDL in our mobile lab for this research project. To assure the quality of the biomarker measurement, the mobile lab protocols were validated three separate times after the start of fieldwork with paired same-subject samples using the same gold-standard commercial laboratory. The quality of the data collected in STAR remained high in each validation sub-study.

These results from validation have important implications for the use of POCTs in field research and global health settings. It is clear that failure to carefully control measurement conditions can compromise the validity of data collected with POCTs that are known to perform well in clinical settings. Measures of HDL and total cholesterol in studies that have not paid attention to controlling measurement conditions are likely to be downward biased, potentially over-stating the prevalence of low HDL in particular (Gaziano et al., 2017, Herningtyas and Ng, 2019).^2^ Our validation establishes that, with adequate attention paid to measurement conditions, POCTs can be successfully deployed to measure biological risks and provide feedback to respondents even in very complex and demanding field environments.

### 3.3 Measurement of cholesterol in the U.S

The NHANES used Mobile-Examination Centers, state-of-the-art facilities enclosed in mobile trailers to measure health biomarkers in the field, including cholesterol biomarkers. The NHANES does regular validation, including third-party contract laboratories that test 2% of all specimens measured, as well as blind split samples validated in labs outside of the Mobile-Examination Centers.

### 3.4 HDL and non-HDL cholesterol: Summary statistics

As shown in Table 1, on average, HDL cholesterol is about 6 mg/dL lower in Indonesia relative to the U.S. for both males and females, which translates into substantially higher rates of low HDL cholesterol (<40 mg/dL) among Indonesians (National Cholesterol Education Program (NCEP) Expert Panel (Adult Treatment Panel III), 2002).^3^ In both countries, HDL is about 10 mg/dL lower for the average male relative to the average female. About half of Indonesian males present with low HDL as opposed to about one-quarter of U.S. males, about 20% of Indonesian females and less than 10% of U.S. females present with low HDL. These are striking differences.

**Table 1:** Summary statistics for Indonesian and American males and females. Means (standard errors) of respondents age 20 and older.

	**Indonesia**		**United States**	
	Males	Females	Males	Females
HDL Cholesterol (mg/dL)	42.0	52.8	48.1	58.7
	(0.3)	(0.4)	(0.4)	(0.4)
% Low HDL (<40 mg/dL)	51.4	21.6	27.1	9.0
	(1.3)	(1.0)	(1.4)	(0.731)
Non-HDL Cholesterol (mg/dL)	148.4	151.0	136.9	134.0
	(0.9)	(1.0)	(1.3)	(1.2)
% High non-HDL (>190 mg/dL)	12.9	16.2	11.0	8.6
	(0.8)	(0.9)	(1.0)	(0.8)
% on medication	0.2	0.3	19.5	16.9
	(0.1)	(0.1)	(1.2)	(1.0)
% High non HDL or on medication	13.0	16.5	29.4	24.3
	(0.6)	(0.7)	(1.4)	(1.2)
Age	40.1	39.9	47.8	49.1
	(0.4)	(0.4)	(0.5)	(0.5)
BMI (kg/m^2^)	23.4	25.4	29.7	30.0
	(0.1)	(0.1)	(0.2)	(0.2)
Waist Circumference (cm)	83.1	85.8	103.5	98.7
	(0.3)	(0.3)	(0.5)	(0.5)
Height (cm)	163.9	151.6	175.1	161.0
	(0.2)	(0.1)	(0.2)	(0.2)
Hours/Week intense physical activity	9.0	2.6	6.7	2.8
	(0.3)	(0.2)	(0.4)	(0.2)
Smoking %	67.2	0.2	18.6	15.1
	(1.2)	(0.1)	(1.2)	(1.0)
ln(Resources per capita) US$	6.5	6.5	10.0	10.0
	(0.0)	(0.0)	(0.0)	(0.0)
Completed level of education (%)				
Middle School	16.5	17.6	4.0	3.6
	(0.9)	(0.9)	(0.3)	(0.3)
Some High School	17.4	15.0	7.9	7.1
	(1.0)	(0.9)	(0.7)	(0.6)
High School Graduate	34.9	25.4	28.4	26.0
	(1.2)	(1.0)	(1.4)	(1.3)
Some College	3.5	3.7	29.1	32.2
	(0.4)	(0.4)	(1.4)	(1.3)
College Graduate or Higher	17.8	22.9	30.5	30.8
	(0.9)	(1.0)	(1.6)	(1.5)
Sample Size	2,689	2,888	2,377	2,560

Notes: Means and (standard errors) stratified by gender for the STAR (Indonesia) and U.S. (NHANES) populations are weighted to reflect the population of Aceh, Indonesia, and the U.S., respectively.

Non-HDL cholesterol levels are higher among Indonesians relative to Americans and in both populations the differences between males and females are small. One of the key challenges in comparing the Indonesian and American non-HDL cholesterol levels has to do with the widespread use of cholesterol-lowering medications in the U.S., in contrast with the very low use of these medications in Indonesia. 18% of Americans in the sample reported using cholesterol-lowering medications, while 0.2% of Indonesians were. Whereas these medications are not designed to affect HDL, they can dramatically lower non-HDL cholesterol levels so that it is difficult to interpret the differences in non-HDL levels across populations.^4^ We define high non-HDL cholesterol as having a measured level >190 mg/dL or being on medication with the assumption that in the absence of medication, the respondent would present with elevated non-HDL. Relative to Indonesians, these rates are considerably higher among Americans than among Indonesians, particularly for males.

In sum, relative to the U.S. population, HDL cholesterol is substantially lower and non-HDL is higher in Indonesia indicating that the Indonesian population has greater cardio-metabolic health risks than Americans. This finding is not unique to the STAR sample. Appendix Table 3 reports parallel data from China, Korea and a different Indonesian sample. While the distributions of cholesterol measures in the Chinese population are similar to those in the U.S., the distributions in the Indonesian and Korean samples are not (they are more similar to the distributions in STAR).

### 3.5 Covariates: Definitions and summary statistics

Table 1 also displays summary statistics of covariates used in the model. The Indonesian sample is younger with an average age of 40 y whereas the average American in the sample is close to 50 y. Three indicators of body composition are included in the models: body mass index (BMI, which is weight in kilograms divided by the square of height in meters), waist circumference (in cms) and height (in cms). Waist circumference is a measure of abdominal adiposity which, in combination with BMI, is linked to elevated cardio-metabolic health risks (Pischon et al., 2008, Ross et al., 2020). Indonesians are shorter, lighter given height and have smaller waists than Americans, which all conventionally suggest lower cardio-metabolic health risks among Indonesians.^5^ But, recall, relative to Americans, Indonesians present with elevated rates of cardio-metabolic risks based on their cholesterol profile.

Physical activity was calculated in Indonesia as the number of hours per week spent doing “intense physical activity.” In the U.S., a question regarding the number of hours per day spent doing “intense physical activity” was used, and multiplied by seven for comparison with the Indonesian measure. In both the U.S. and Indonesia, females report 2.5–3 hours per week of physical activity, on average, whereas American males report about twice as much activity and Indonesian males about 3 times as much physical activity. An indicator variable for whether or not an individual was a current smoker was used to control for smoking behavior. Over two-thirds of Indonesian males are smokers and very few females smoke; about 1 in 6 Americans smoke and the gender gap is relatively small.

In the U.S., household income is thought to be a good measure of resources and we use the ln(income per capita), lnPCY, for the models with the NHANES data. In low-income countries, income fluctuates substantially over time and consumption is thought to be a better measure of resource availability. STAR therefore collects detailed information on household consumption which cover expenditures and the imputed value of own production and, combining the two, we construct ln(per capita household expenditure), lnPCE. They are displayed in USD in Table 1: on average, American households have about 30 times as much as Indonesian households. In terms of purchasing power parity, Americans spend about 10 times as much as Indonesians. Paralleling these comparisons, Americans have completed more education than Indonesians: over half of Americans have some college but only a quarter of Indonesians have achieved that level.

## 4 Results

Before turning to comparisons based on multivariable regression models, we compare the Indonesian cholesterol profiles for males and females with a series of subsamples of Americans using the NHANES data.

### 4.1 Comparing cholesterol profiles in Indonesia and the U.S

Rows 1 and 3 of Table 2 repeat the results in Table 1 for Indonesians and Americans, respectively, and we also display the gender gaps for each population. The male-female gaps in levels are similar for HDL, but reversed for non-HDL cholesterol indicating that, relative to females, American males have worse non-HDL but Indonesian males have better non-HDL cholesterol. The same conclusions are drawn for HDL and non-HDL cut-offs that indicate elevated risks. Accounting for the different age structure of the Indonesian population by re-weighting the Indonesian sample to reflect the NHANES population in row 2 of the table, the between-country disparities expand, and the Indonesian gender gap in non-HDL cholesterol gets even larger (+2.9 mg/dL in the U.S., -7.2 mg/dL in Indonesia).

**Table 2:** Lipid Profiles in Indonesia and U.S. Means (standard errors) of respondents age 20 and older.

		**A. HDL Cholesterol**							**B. Non-HDL Cholesterol**					
		Level (mg/dL)			%<40			Level (mg/dL)				%>190 | medication		
		Males	Females	M-F Gap	Males	Females	M-F Gap	Males		Females	M-F Gap	Males	Females	M-F Gap
Indonesia (STAR)														
	1. Unadjusted	42.0	52.8	-10.8	51.4	21.6	29.8	148.4		151.0	-2.6	13.0	16.5	-3.4
		(0.3)	(0.4)	(0.5)	(1.3)	(1.0)	(1.6)	(0.9)		(1.0)	(1.3)	(0.8)	(0.9)	(1.2)
													
	2. Age adjusted	43.3	53.8	-10.5	47.4	20.3	27.1	152.2		159.4	-7.2	16.6	22.3	-5.7
	(same as U.S.)	(0.4)	(0.4)	(0.5)	(1.3)	(1.0)	(1.7)	(1.1)		(1.1)	(1.6)	(1.0)	(1.1)	(1.5)
U.S. (NHANES)														
	3. All U.S.	48.1	58.7	-10.6	27.1	9.0	18.0	136.9		134.0	2.9	29.4	24.3	5.1
		(0.4)	(0.4)	(0.6)	(1.4)	(0.731)	(1.6)	(1.3)		(1.2)	(1.7)	(1.4)	(1.2)	(1.9)
	4. Asian ethnicity	46.6	59.9	-13.3	31.7	6.5	25.3	145.5		132.3	13.2	37.3	20.4	16.9
		(0.8)	(0.9)	(1.2)	(3.0)	(1.3)	(3.2)	(2.9)		(2.1)	(3.6)	(3.0)	(2.1)	(3.6)
	5. Asian immigrants	46.4	59.4	-12.9	32.3	7.4	25.0	147.4		133.7	13.6	40.6	22.3	18.3
		(0.9)	(0.9)	(1.3)	(3.2)	(1.5)	(3.5)	(3.2)		(2.3)	(4.0)	(3.2)	(2.3)	(4.0)
	6. Low income	45.6	55.0	-9.4	32.8	13.2	19.6	138.2		133.5	4.6	29.4	19.1	5.3
		(0.6)	(0.6)	(0.9)	(2.4)	(1.5)	(2.8)	(2.3)		(1.7)	(2.9)	(2.2)	(1.7)	(2.7)
	7. Low education	45.6	52.7	-7.0	37.7	17.4	20.2	141.9		137.1	4.7	31.1	28.7	2.3
		(0.7)	(0.8)	(1.0)	(3.1)	(2.5)	(4.0)	(2.8)		(2.7)	(3.9)	(2.8)	(2.8)	(4.0)
Sample sizes														
	Indonesia	2,689	2,888		2,689	2,888		2,689		2,888		2,689	2,888	
	All U.S.	2,377	2,560		2,377	2,560		2,377		2,560		2,377	2,560	
	Asian ethnicity	320	374		320	374		320		374		320	374	
	Asian immigrants	275	331		275	331		275		331		275	331	
	Low income	682	823		682	823		682		823		682	823	
	Low education	514	479		514	479		514		479		514	479	

Notes: Coefficients and standard error for proportion over cutoffs multiplied by 100 to represent percentages. Age-adjustments reflect re-weighting of the Indonesia sample in 5-year age groups to match NHANES taking into account self-identified race and immigration status. In the U.S., low income is defined as being in the lowest 1/3 of the per capita income distribution, and low education is defined as those with less than a high school diploma. Heteroscedasticity-consistent robust standard errors in parentheses.

Rows 4 and 5 of Table 2 restrict attention to Asian ethnicity and Asian-immigrant individuals in the NHANES population, respectively. HDL cholesterol levels are slightly lower for males and slightly higher for females relative to the entire population but remain substantially higher than those measured in Indonesia. While the non-HDL cholesterol levels for Asian men living in the U.S. are similar to Indonesian men, Indonesian women have much higher non-HDL cholesterol than Asian women in the U.S., and the inverted non-HDL cholesterol gender disparity in the Indonesian population is not observed among Asian individuals living in the U.S. The non-HDL cholesterol gender difference appears to be primarily driven by very high levels of non-HDL cholesterol among Indonesian females.

Indonesians are not only younger and of a different ethnicity but, as shown in Table 1, they are lower income and have less education. The final two rows of Table 2 restrict attention to Americans with low income (i.e. in the bottom tercile of the PCY distribution) in row 6, and lower education (i.e. less than a high school diploma) in row 7. Patterns are broadly similar when examining these sub-groups relative to all Americans. HDL cholesterol levels among men are still higher in the U.S. than in Indonesia, though among women HDL cholesterol levels among the low-income and low-education populations are more similar to those in Indonesia. Nonetheless, more men and women in Indonesia fall below the clinically-relevant low cutoff for HDL than low SES groups in the U.S. With respect to non-HDL cholesterol, levels are slightly higher than the rest of the American population but remain substantially lower than among Indonesians, and the gender gap is still the opposite sign among low SES Americans when compared to Indonesians.

Neither adjusting for age differences between Indonesians and Americans, nor focusing on subsets such as those of Asian ethnicity and those with less income or education, can explain the differences in cholesterols across the populations. Nor can these efforts account for the distinctive gender patterns in Indonesia. We turn in the next sub-section to multivariable regression models to further explore the roles that observed characteristics play in driving the differences between the U.S. and Indonesian populations.

### 4.2 Comparing predictors of cholesterol profiles in Indonesia and the U.S

Estimates of [2] and [3] are reported in Table 3 for HDL cholesterol and in Table 4 for non-HDL cholesterol. In each panel, estimates of [2] are reported for Indonesian and American respondents in the first two columns and the third column reports the interaction terms in [3] which are the differences between the estimates for Indonesians relative to Americans. In that column, the intercept reflects the adjusted difference between Americans and Indonesians. The estimates are stratified by gender. In each table, panel A reports results for levels of cholesterol and panel B reports results for the cut-off indicating elevated risk. All models are estimated using OLS with coefficient estimates reported above standard errors; F test statistics for joint significance are reported at the bottom of each column, for all covariates included in the country-specific models and for the joint significance of the differences between those coefficients in the third column in each panel. All variance and covariance estimates take into account arbitrary heteroscedasticity (Huber, 1967).^6^

**Table 3:** Multivariable regression models of HDL cholesterol by country and gender.

	**A. HDL Cholesterol Level (mg/dL)**						**B. HDL Cholesterol<40 %**					
	Indonesian Males	U.S. Males	Difference (Indo-U.S.)	Indonesian Females	U.S. Females	Difference (Indo-U.S.)	Indonesian Males	U.S. Males	Difference (Indo-U.S.)	Indonesian Females	U.S. Females	Difference (Indo-U.S.)
Indicator variable if												
- Age 20-29y	-0.71	-0.34	-0.36	-0.28	-0.32	0.04	-2.96	-1.28	-1.68	2.62	-2.40	5.02
	(0.96)	(1.11)	(1.47)	(0.93)	(1.16)	(1.48)	(3.50)	(3.96)	(5.28)	(2.64)	(2.59)	(3.70)
- Age 40-50y	0.14	0.60	-0.47	1.06	2.24	-1.18	-1.19	0.74	-1.93	-1.80	-6.80	5.00
	(0.98)	(1.20)	(1.55)	(1.04)	(1.29)	(1.66)	(3.49)	(4.45)	(5.65)	(3.00)	(2.36)	(3.81)
- Age 50-60y	1.20	3.31	-2.11	0.37	4.84	-4.46	-9.98	-1.51	-8.47	1.76	-5.44	7.20
	(1.14)	(1.40)	(1.81)	(1.15)	(1.34)	(1.77)	(4.36)	(4.43)	(6.22)	(3.45)	(2.79)	(4.44)
- Age 60-70y	4.34	0.84	3.50	1.99	4.75	-2.75	-15.79	2.86	-18.65	0.19	-6.94	7.13
	(1.55)	(1.24)	(1.98)	(1.56)	(1.54)	(2.19)	(5.08)	(4.91)	(7.07)	(4.25)	(2.46)	(4.91)
- Age 70+ y	2.35	3.84	-1.49	1.45	7.42	-5.97	-10.08	-6.53	-3.55	5.81	-7.95	13.76
	(2.28)	(1.24)	(2.59)	(2.04)	(1.37)	(2.46)	(7.16)	(4.65)	(8.54)	(5.69)	(2.64)	(6.27)
BMI	-0.38	-0.31	-0.07	-0.60	-0.07	-0.52	1.45	0.81	0.64	0.89	0.04	0.84
	(0.16)	(0.11)	(0.20)	(0.13)	(0.11)	(0.16)	(0.59)	(0.45)	(0.74)	(0.35)	(0.18)	(0.39)
Waist Circum. (cm)	-0.11	-0.17	0.06	-0.08	-0.27	0.18	0.39	0.39	0.00	0.25	0.31	-0.05
	(0.06)	(0.04)	(0.08)	(0.06)	(0.05)	(0.07)	(0.22)	(0.18)	(0.29)	(0.15)	(0.09)	(0.17)
Height (cm)	0.00	0.02	-0.02	-0.08	0.12	-0.21	0.08	-0.02	0.10	0.26	0.27	-0.01
	(0.06)	(0.05)	(0.08)	(0.07)	(0.07)	(0.10)	(0.22)	(0.19)	(0.29)	(0.20)	(0.13)	(0.23)
Physical Activity	-0.03	0.10	-0.13	-0.01	-0.02	0.01	0.05	-0.20	0.24	-0.09	0.09	-0.18
	(0.02)	(0.04)	(0.04)	(0.06)	(0.04)	(0.07)	(0.09)	(0.09)	(0.13)	(0.11)	(0.10)	(0.15)
Indicator var if	-1.39	-0.78	-0.60	5.03	-3.04	8.07	7.33	3.73	3.60	-17.48	2.38	-19.86
Currently smoker	(0.74)	(1.09)	(1.31)	(2.65)	(1.06)	(2.86)	(2.75)	(3.42)	(4.39)	(4.60)	(2.21)	(5.11)
ln(PCE or PCY)	0.43	0.88	-0.45	1.62	1.00	0.63	-1.47	-4.86	3.39	-4.91	-1.33	-3.58
in U.S.$	(0.58)	(0.37)	(0.69)	(0.64)	(0.43)	(0.77)	(2.13)	(1.34)	(2.52)	(1.84)	(0.75)	(1.99)
Indicator var for completed education												
- Middle School	-0.14	-3.03	2.89	-0.28	-3.37	3.09	2.37	6.53	-4.15	-0.53	5.75	-6.28
	(1.05)	(1.37)	(1.73)	(1.02)	(1.24)	(1.61)	(3.71)	(4.71)	(6.00)	(2.83)	(3.65)	(4.61)
- Some High School	0.34	-2.02	2.36	-0.67	-2.50	1.83	2.43	8.63	-6.21	3.87	7.67	-3.80
	(1.07)	(1.20)	(1.61)	(1.12)	(1.17)	(1.62)	(3.57)	(4.70)	(5.90)	(3.35)	(3.61)	(4.93)
- Some College	0.41	0.37	0.04	-0.17	2.66	-2.83	1.06	-3.01	4.07	0.02	-1.46	1.48
	(1.80)	(0.97)	(2.04)	(1.46)	(1.02)	(1.78)	(6.77)	(3.40)	(7.58)	(4.92)	(2.04)	(5.32)
- College	0.65	1.33	-0.68	1.52	3.38	-1.86	2.08	-1.35	3.43	-2.41	-3.56	1.16
	(0.89)	(1.14)	(1.44)	(0.93)	(1.27)	(1.58)	(3.50)	(4.04)	(5.34)	(2.62)	(2.13)	(3.38)
Indicator var if	2.36	-1.49	3.85	3.85	-2.12	5.97	22.96	3.34	19.61	-7.99	4.07	-12.06
on cholesterol medication	(5.32)	(1.06)	(5.43)	(4.78)	(1.18)	(4.92)	(22.82)	(3.84)	(23.14)	(13.42)	(2.38)	(13.63)
Intercept	40.66	47.49	-6.83	52.28	54.82	-2.54	55.37	30.48	24.89	18.99	14.38	4.61
	(1.16)	(1.33)	(1.76)	(1.43)	(1.23)	(1.89)	(4.52)	(4.66)	(6.50)	(4.01)	(2.61)	(4.78)
Observations	2,689	2,377		2,888	2,560		2,689	2,377		2,888	2,560	
F statistic for joint signficance	4.85	15.34	12.79	9.48	22.02	16.82	5.61	9.06	15.90	4.99	6.29	10.74
(p value)	(0.000)	(0.000)	(0.000)	(0.000)	(0.000)	(0.000)	(0.000)	(0.000)	(0.000)	(0.000)	(0.000)	(0.000)

Notes: See Notes to Table 3A. Coefficients multiplied by 100.

**Table 4:** Multivariable regression models of non-HDL cholesterol by country and gender.

	**A. Non-HDL Cholesterol Levels (mg/dL)**						**B. Non-HDL Cholesterol>190 or Cholesterol Medication**					
	Indonesian Males	U.S. Males	Difference (Indo-U.S.)	Indonesian Females	U.S. Females	Difference (Indo-U.S.)	Indonesian Males	U.S. Males	Difference (Indo-U.S.)	Indonesian Females	U.S. Females	Difference (Indo-U.S.)
Indicator variable if												
- Age 20-29y	-8.13	-17.28	9.15	-7.72	-10.27	2.55	0.22	-2.94	3.17	-3.49	-1.17	-2.32
	(2.38)	(3.96)	(4.62)	(2.49)	(3.13)	(3.99)	(1.95)	(3.57)	(4.07)	(2.00)	(2.04)	(2.86)
- Age 40-50y	8.99	4.79	4.20	9.13	9.54	-0.41	4.01	2.21	1.80	4.76	2.73	2.03
	(2.43)	(3.79)	(4.50)	(2.62)	(3.23)	(4.16)	(2.21)	(4.36)	(4.89)	(2.71)	(2.24)	(3.51)
- Age 50-60y	13.07	1.25	11.82	20.32	24.96	-4.65	5.31	12.52	-7.21	14.20	23.76	-9.56
	(2.62)	(4.36)	(5.09)	(3.26)	(3.88)	(5.07)	(2.95)	(4.61)	(5.47)	(3.39)	(3.60)	(4.95)
- Age 60-70y	7.95	-7.68	15.63	27.61	23.54	4.07	6.17	25.05	-18.88	16.22	39.34	-23.12
	(3.74)	(4.28)	(5.68)	(4.59)	(3.92)	(6.03)	(3.85)	(5.20)	(6.47)	(4.68)	(4.31)	(6.37)
- Age 70+ y	7.38	-15.82	23.20	15.23	11.10	4.13	0.27	37.71	-37.44	10.16	42.38	-32.22
	(4.72)	(4.50)	(6.52)	(5.18)	(4.00)	(6.55)	(4.45)	(4.77)	(6.53)	(5.44)	(3.67)	(6.56)
BMI	2.16	0.40	1.76	0.54	-0.86	1.39	0.70	-0.10	0.81	-0.17	-0.49	0.31
	(0.37)	(0.33)	(0.50)	(0.35)	(0.27)	(0.44)	(0.34)	(0.50)	(0.60)	(0.32)	(0.28)	(0.42)
Waist Circum. (cm)	0.34	0.33	0.01	0.60	0.66	-0.06	0.47	0.39	0.08	0.50	0.40	0.09
	(0.14)	(0.14)	(0.20)	(0.15)	(0.13)	(0.20)	(0.12)	(0.21)	(0.24)	(0.14)	(0.14)	(0.20)
Height (cm)	-0.38	-0.56	0.18	-0.05	-0.16	0.11	-0.42	-0.32	-0.09	-0.04	-0.06	0.02
	(0.15)	(0.18)	(0.23)	(0.18)	(0.17)	(0.25)	(0.15)	(0.19)	(0.24)	(0.17)	(0.17)	(0.24)
Physical Activity	-0.15	-0.17	0.02	0.08	-0.02	0.10	-0.09	-0.16	0.07	0.03	0.07	-0.04
	(0.06)	(0.11)	(0.12)	(0.13)	(0.13)	(0.19)	(0.04)	(0.10)	(0.11)	(0.14)	(0.12)	(0.18)
Indicator var if	1.84	1.12	0.72	0.10	6.43	-6.33	-1.06	0.91	-1.97	4.84	2.66	2.18
Currently smoker	(1.91)	(2.67)	(3.29)	(13.06)	(3.25)	(13.46)	(1.80)	(3.29)	(3.75)	(14.08)	(2.98)	(14.39)
ln(PCE or PCY)	0.35	2.44	-2.09	-3.06	0.53	-3.60	0.60	3.17	-2.57	-0.94	1.64	-2.58
in U.S.$	(1.46)	(1.29)	(1.95)	(1.69)	(1.28)	(2.12)	(1.35)	(1.31)	(1.88)	(1.62)	(1.24)	(2.04)
Indicator var for completed education												
- Middle School	0.22	5.38	-5.16	0.84	10.58	-9.74	2.56	4.18	-1.62	-0.98	4.69	-5.67
	(2.31)	(4.48)	(5.04)	(2.49)	(5.37)	(5.92)	(2.23)	(4.70)	(5.20)	(2.64)	(4.65)	(5.35)
- Some High School	-0.55	4.72	-5.27	2.63	-0.20	2.83	0.79	1.16	-0.38	-0.26	-0.84	0.58
	(2.47)	(3.89)	(4.60)	(3.07)	(3.73)	(4.83)	(2.17)	(4.51)	(5.00)	(2.74)	(4.15)	(4.97)
- Some College	1.79	-2.01	3.80	1.53	-0.57	2.09	1.77	-2.27	4.04	-2.38	-2.90	0.52
	(3.99)	(3.06)	(5.03)	(4.53)	(2.95)	(5.41)	(4.79)	(3.34)	(5.83)	(3.63)	(3.13)	(4.79)
- College	-0.77	-3.63	2.86	1.10	-0.09	1.19	1.60	0.09	1.51	-0.11	-8.70	8.59
	(2.49)	(3.62)	(4.39)	(2.41)	(3.60)	(4.33)	(2.35)	(4.10)	(4.73)	(2.13)	(3.70)	(4.27)
Indicator var if	-1.07	-24.70	23.63	-3.91	-20.49	16.58						
on cholesterol medication	(21.61)	(3.53)	(21.90)	(8.25)	(3.59)	(8.99)						
Intercept	158.14	146.39	11.74	145.42	123.96	21.46	19.94	13.35	6.59	14.66	6.12	8.54
	(3.07)	(4.44)	(5.40)	(3.72)	(3.35)	(5.00)	(2.84)	(4.82)	(5.59)	(3.53)	(3.16)	(4.74)
Observations	2,689	2,377		2,888	2,560		2,689	2,377		2,888	2,560	
F statistic for joint signficance	22.72	15.09	21.00	21.26	15.52	24.04	8.05	18.52	17.58	8.84	28.64	18.14
(p value)	(0.000)	(0.000)	(0.000)	(0.000)	(0.000)	(0.000)	(0.000)	(0.000)	(0.000)	(0.000)	(0.000)	(0.000)

Notes: See Notes to Table 3B.

#### 4.2.1 Age

Age is specified flexibly with indicator variables for each 10 y age group in the regression models; the excluded group is people aged 30–39 y. As shown in panel A of Table 3, among males, HDL tends to rise with age for both Indonesians and Americans and the differences in the profiles are not statistically significant. The gradients for males translate into significantly lower rates of HDL below the 40 mg/dL cutoff for older Indonesian males but not for American males which is a reflection of the higher HDL levels among Americans (panel B). HDL also rises with age among females and the age gradient is steeper among American females so older Indonesian females have lower levels of HDL and are also more likely to present with HDL below the 40 mg/dL cut-off.

**Fig. 2:**
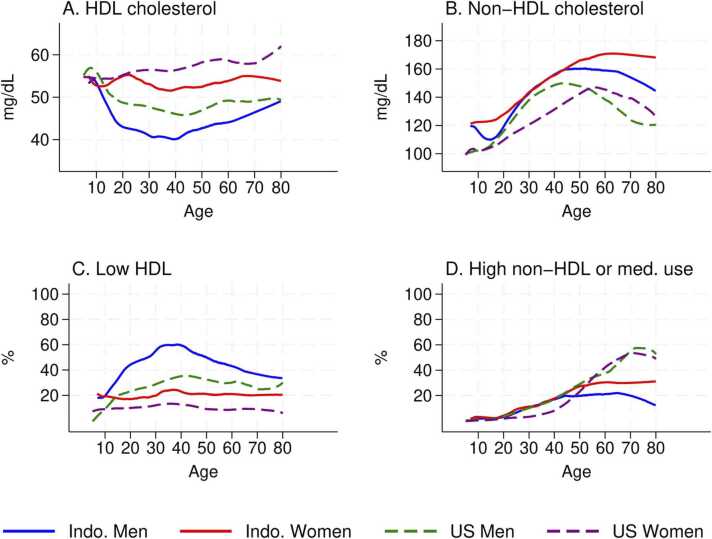
Cholesterol, age, and population.

A visual summary of the age profiles is provided by Fig. 2 which displays bivariable non-parametric estimates of the relationship between cholesterol and age separately for Indonesian and American males and females (Cleveland, 1981).^7^ The estimates include respondents of all ages in the samples in contrast with all other analyses which are restricted to those aged 20 y and older in order to illustrate an important point: at young ages, differences between the groups are very small. Fig. 2A displays the relationships between HDL cholesterol and age: for children aged <12 y, there are no differences in HDL between males and females and the HDL differences between Indonesians and Americans are small. The gender gaps in both populations emerge around age 15 y when males’ HDL declines substantially until age 40 y in both the U.S. and Indonesia. There is no parallel decline among females in either population. Similar patterns are observed in the rates of low HDL (Fig. 2C) and rates are higher among Indonesians reflecting the entire distribution of HDL is shifted to the left for Indonesians relative to Americans.

Table 4 reports results for non-HDL cholesterol which increases with age for all Indonesians, especially females, before declining slightly at the oldest ages. Among Americans, it rises among young males and females and then declines significantly at older ages, particularly among American males (panel A). As a result, there are large differences in the gradients for Indonesian males relative to Americans but no differences between American and Indonesian females. These patterns in non-HDL cholesterol are not straightforward to interpret since over 15% of Americans are on cholesterol-lowering medications whereas <0.5% of Indonesians report medication use. In panel B of the table, we report the probability a respondent presents with high non-HDL cholesterol (>190 mg/dL) or reports using medication. These rates rise dramatically with age for all Americans which, comparing the gradients with measured non-HDL, reflects the role of medication. The rates also increase for Indonesian females, but are much lower than for U.S. females. Rates do not rise with age for Indonesian males. As a result, there are large and significant differences in the gradients for older Americans (age>50 y) relative to Indonesians. These patterns are visually summarized in panels B and D of Fig. 2 which underscores the very high levels of non-HDL cholesterol among older Indonesians, relative to Americans, but the much higher rates of elevated non-HDL or medication among older Americans. The high levels of non-HDL cholesterol that are untreated in the Indonesian population is an important concern that has implications for the future trajectory of cardiovascular disease, population health and health care costs.

#### 4.2.2 Anthropometry and physical activity

The models include three anthropometric measures, BMI, waist circumference and height, along with self-reported hours of physical activity. Holding height constant, variation in BMI largely reflects variation in weight and holding both constant, waist circumference is indicative of visceral fat.

HDL declines with BMI and waist circumference for both American and Indonesian males at about the same rate; both are also significant predictors of higher rates of low HDL. The patterns for females are different. Among Indonesian females, HDL declines significantly as BMI increases, but not as waist circumference increases, and among American females waist circumference is a significant predictor of HDL but BMI is not. The differences in these gradients between American and Indonesian females are significant. Parallel patterns are apparent for the probability that HDL is low.^8^ Height is a significant predictor but only for American females and only for the probability that HDL is below the cut-off.

Abdominal fat is important for non-HDL cholesterol which increases significantly with waist circumference for all four populations. The estimates are the same for Indonesians and Americans but they are much larger for females in both populations. The estimates are also positive for high non-HDL but there is no difference between males and females. The relationships with BMI are more nuanced, with BMI being a significant positive predictor of non-HDL among Indonesian males but a negative predictor for American females; the latter is not entirely driven by higher medication use among heavier American females as the association is also negative for high non-HDL (and medication use) although that estimate is not statistically significant. Among males, height is protective for both Americans and Indonesians but there is no link with height among females.

**Fig. 3:**
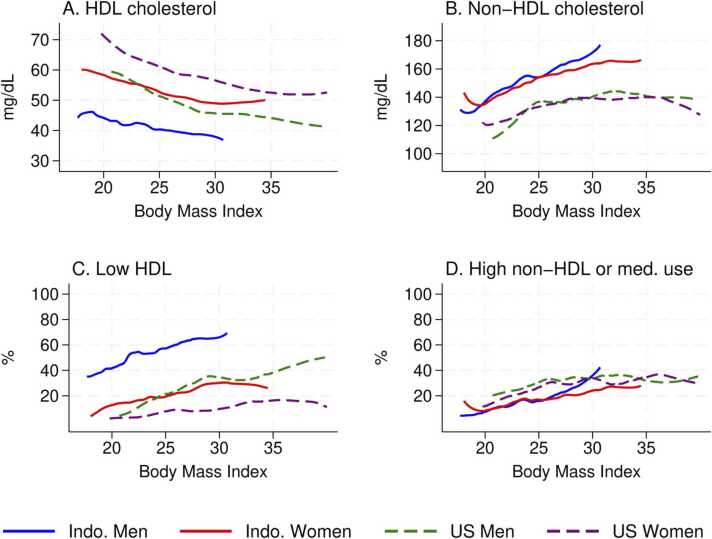
Cholesterol, BMI, and population.

The distinct patterns of the relationships of cholesterol with BMI and waist circumference across the Indonesian and U.S. populations suggest that the markers have different interpretations for cardiovascular disease risks in the two contexts. Since Indonesians are lighter, given height, and have smaller waist circumferences, the different patterns may reflect differences in levels of the anthropometric measures across the populations and non-linear relationships between cholesterol and anthropometry. That does not appear to be the case. For example, Fig. 3 displays non-parametric estimates of the relationship between the cholesterol markers and BMI for each sample. There is considerable overlap in the distributions of BMI and the relationships are roughly linear. HDL cholesterol levels are higher for Indonesian males and females, relative to Americans, across the entire BMI distribution. Non-HDL cholesterol is more complicated to interpret as the levels are higher among Indonesians but that may be because medication rises with BMI among Americans and so the probability that non-HDL is high or the respondent is on medication is essentially the same for all four groups, conditional on BMI.

Conditional on these anthropometric measures, physical activity is not a significant predictor of either cholesterol for females. Among American males, those who participate in more physical activity have significantly higher HDL and lower non-HDL (which is only significant at 10%). Relative to Americans, Indonesian males are far more active and those who are more physically active have significantly lower non-HDL cholesterol but there is no significant relationship with HDL.

#### 4.2.3 Smoking

Smoking is of particular interest because it has been consistently linked to adverse cholesterol biomarkers. Over two-thirds of Indonesian males smoke – which is much higher than rates among American males and females – while almost no Indonesian females smoke. Americans who smoke have lower HDL and higher non-HDL; the effect sizes are over three times larger for females and significant. Among Indonesian males, smoking is significantly associated with lower HDL and higher rates of low HDL but is not related to non-HDL cholesterol.

#### 4.2.4 Socioeconomic characteristics and medication use

To take into account variation in socioeconomic characteristics, the models include completed education and measures of household resources – lnPCE for Indonesia and lnPCY for the U.S. Education is not related to any of the cholesterol indicators in any of the populations. Household resources are associated with better cholesterol outcomes for Americans with the effect sizes typically being substantially larger for males than females but there are no links for Indonesians. For both education and resources, there are no significant differences in the gradients between the two countries which is remarkable given the very large differences in levels of socioeconomic status.^9^ Finally, Americans on medication have far lower levels of non-HDL but HDL is unaffected; both of these results are expected. The very low rates of medication in Indonesia preclude providing informative estimates of those relationships.

#### 4.2.5 Joint significance of covariates

The F test statistics at the bottom of each country-specific model establish that, taken together, the covariates are significant predictors of each cholesterol marker. The F test statistics at the bottom of the difference columns establish that the differences are also jointly significant in every model. We turn next to investigate the specific factors that underlie the differences across the countries and then explore the differences between males and females in Indonesia.

### 4.3 Differences across countries

Table 5 investigates the differences in HDL and non-HDL cholesterol between the two countries separately for males and females. Evidence for HDL cholesterol is reported in panel A, for levels and low HDL. The upper panel documents how the gap between Indonesians and Americans varies as we add regression controls. The first row displays the large unadjusted differences for both males and females, which shrink by about 10 percent for HDL and by about 5 percent for HDL<40 mg/dL after adjusting for age. The key point, however, is that the adjusted gaps are larger than the unadjusted gaps after taking into account differences in BMI, education, smoking and medication use, all of which have been shown to be predictive of cholesterol.

**Table 5:** Differences between Indonesian and US males and females adjusting for observed differences. Coefficients (standard errors) unadjusted and adjusting for observed characteristics.

**A. HDL Cholesterol**								
	A1. Level (mg/dL)				A2. % HDL<40 mg/dL			
Difference (Indo-U.S.)	Males		Females		Males		Females	
	[1]		[2]		[3]		[4]	
Unadjusted	-6.08		-5.84		24.36		12.58	
	(0.54)		(0.57)		(1.86)		(1.25)	
Adjusted for age	-5.51		-5.30		23.55		11.92	
	(0.54)		(0.57)		(1.88)		(1.31)	
Adjusted for age, BMI, education	-8.17		-7.82		28.00		17.13	
smoking, and medication	(0.65)		(0.59)		(2.04)		(1.43)	
Oaxaca-Blinder Decomposition	Explained	Unexplained	Explained	Unexplained	Explained	Unexplained	Explained	Unexplained
Age	-0.84	0.56	0.35	-2.63	4.99	-9.00	-1.80	7.57
BMI	3.21	0.81	3.93	-1.00	-17.63	4.93	-9.00	4.96
Smoking	-0.92	0.16	-1.93	2.46	4.53	-0.64	5.67	-6.28
Medication Use	-1.30	1.66	-1.61	2.00	-8.02	7.34	3.07	-3.78
Education	0.63	-0.73	1.33	-3.22	-3.92	6.28	0.20	0.51
Intercept		-9.31		-5.53		35.51		11.46
Sum:	0.78	-6.85	2.07	-7.92	-20.05	44.42	-1.86	14.44

**B. Non-HDL Cholesterol**								
	B1. Level (mg/dL)				B2. % Non-HDL>190 mg/dL or on medication			
Difference (Indo-U.S.)	Males		Females		Males		Females	
	[1]		[2]		[3]		[4]	
Unadjusted	11.50		16.99		-16.38		-7.82	
	(1.58)		(1.53)		(1.64)		(1.51)	
Adjusted for age	13.80		19.97		-10.37		-1.03	
	(1.56)		(1.47)		(1.58)		(1.40)	
Adjusted for age, BMI, education	15.94		21.75		-5.95		1.87	
smoking, and medication	(1.79)		(1.65)		(1.83)		(1.57)	
Oaxaca-Blinder Decomposition	Explained	Unexplained	Explained	Unexplained	Explained	Unexplained	Explained	Unexplained
Age	-6.31	12.84	-6.19	0.22	3.86	-13.56	1.60	-16.16
BMI	-29.84	16.25	-12.78	8.89	-17.05	8.75	-5.39	3.36
Smoking	1.83	-0.68	0.47	-1.48	-0.86	0.12	-1.33	0.95
Medication Use	-4.86	9.50	-3.65	7.01	-	-	-	-
Education	-3.28	3.75	-0.99	1.81	-1.58	2.07	-0.31	3.33
Intercept		12.33		23.66		1.88		6.12
Sum:	-42.46	53.99	-23.1	40.1	-15.63	-0.74	-5.43	-2.40

Notes: Estimates of the difference in levels between Indonesia and the US are presented unadjusted and controlled. Age-adjustments control semi-parametrically for age with a series of age indicator variables by decade. BMI is a continuous variable, and smoking and medication are binary indicators. Medication use controlled in model B1. Respondents age 20 or older. Coefficients for proportion over cutoffs multiplied by 100 to represent percentages. Heteroscedasticity-consistent robust standard errors used.

This is explored further in the lower panel of the table which reports Blinder-Oaxaca decompositions that separate, for each covariate, the part that can be attributed to differences in the observed characteristic, given the U.S. coefficient estimates (explained part) and the part that is attributable to differences in coefficient estimates across the two countries (unexplained part). BMI, smoking and medication use play a dominant role in both the explained and unexplained components. Moreover, the explained and unexplained decompositions are typically in opposite directions suggesting that the underlying production functions may be different in the populations.

Evidence for non-HDL cholesterol is displayed in panel B of the table. Indonesians have higher levels of non-HDL than Americans. Adjusting for age increases the gaps in levels and including all the controls increases the gaps even further so they are about 50 percent larger than the unadjusted gaps. The Blinder-Oaxaca decompositions indicate that BMI plays a dominant role in both the explained and unexplained although age, education and medication use are also important.

In contrast, Indonesians are far less likely to present with high non-HDL cholesterol or be on medication because medication use is much higher among Americans. Adjusting for characteristics, the gaps are reduced to less than one-third of the unadjusted gaps and are not significant for females. The Blinder-Oaxaca decompositions indicate differences in BMI across the populations are dominant in the explained differences and age is a major factor in the unexplained differences.

### 4.4 Differences between male and female Indonesians

As discussed above, and shown in Table 2, Indonesian and American males have substantially and significantly lower HDL cholesterol relative to females. The gap is about 10 mg/dL which translates into much higher rates of low HDL among males. These differences between males and females in the two populations are repeated in the first two rows of the left-hand panel of Table 6. In sharp contrast, as shown in the right-hand panel, relative to females, American males have higher levels of non-HDL cholesterol but in Indonesia this pattern is reversed with males having lower non-HDL cholesterol than females. To investigate this inverted cholesterol epidemiology in Indonesia relative to the U.S., we delve further into the gender differences in Indonesia by exploiting the fact that data are collected from multiple people within households (Frankenberg et al., 2016, Moghani Lankarani et al., 2017, Vogel et al., 2021).

**Table 6:** Gender differences: Within household comparisons in Indonesia. Male-female differences (standard errors).

	**A. HDL Cholesterol**		**B. Non-HDL Cholesterol**	
	HDL	% HDL<40	Non-HDL	%Non-HDL >190 or Med
A. United States	-10.6	18.0	2.9	5.1
	(0.6)	(1.4)	(1.7)	(1.9)
B. Indonesia				
1. Uncontrolled differences				
Male-Female	-10.8	29.8	-2.6	-3.4
	(0.5)	(1.4)	(1.3)	(0.9)
Difference from US Gender Gap	*0.2*	*-11.8*	*5.5*	*8.5*
2. Control shared household characteristics only				
Male-Female	-11.0	31.5	0.5	-1.3
	(0.4)	(1.3)	(1.1)	(1.1)
3. Also include all controls interacted with gender				
Male-Female	-11.4	32.5	11.2	5.5
	(1.5)	(4.7)	(3.9)	(3.7)
Observations	5,577	5,577	5,577	5,577
# of Households	3,108	3,108	3,108	3,108

Notes: Coefficients for proportion over cutoffs multiplied by 100 to represent percentages. Uncontrolled models parallel Table 2 and use heteroscedasticity-consistent standard errors. Standard errors from models with household fixed effects are robust to heteroscedasticity and clustered at level of household. All covariates listed in Tables 3 and 4 included in model in B.3.

These differences may reflect unobserved factors that influence blood lipids including, for example, diet (which is not measured in the survey) and, relatedly, individual-specific resource allocation. For example, the empirical models described above take into account individual-level anthropometry, physical activity, and education, which are likely to be related to diet and individual-level resources, as well as household-level resources. Those resources may be allocated differently by gender reflecting, for example, cultural norms, individual-specific needs and possibly returns to the allocation in terms of greater earnings by the individual. The allocations may also reflect differences in bargaining power within the household. to (Haddad et al., 1997, Hoddinott and Haddad, 1995, Strauss and Thomas, 1995, Thomas, 1990). These forces may play a unique role in determining differences in the non-HDL gender epidemiology in Indonesia.

Results from estimating model [4], including household fixed effects, are reported in the rest of Table 6. Variance and covariance estimates take into account heteroscedasticity and clustering within households. Results that only adjust for the household are reported in panel B.2. The gender gaps in HDL are little changed. However, the non-HDL gaps are essentially reduced to zero. In panel B.3, the model includes all individual-specific covariates along with all individual- and household-specific covariates interacted with gender. Again, the gender gaps in HDL are little changed but the gaps for non-HDL become large and positive, reversing the signs of the unadjusted gaps. The gap for non-HDL levels is far larger than the gap in the U.S. and the gap for elevated non-HDL (or medication) is very similar to the U.S. gap. These results indicate that the gender gaps in HDL are likely to reflect biological differences across the genders but the gaps in non-HDL cholesterol also reflect differences in behaviors and resource allocation within households in Indonesia.^10^ The results suggest there are important differences between Indonesia and the U.S., particularly with regards to gender roles, related to unmeasured behavioral, socioeconomic and environmental factors – and in all likelihood, interactions between the three.

## 5 Conclusion

Indonesians present with adverse lipid profiles, relative to Americans: both male and female Indonesians have lower levels of HDL cholesterol and higher levels of non-HDL cholesterol than male and female Americans, respectively. This is surprising since the Indonesian population is younger, has lower BMI and less visceral fat, which are three major correlates of adverse cholesterol levels. Furthermore, American females have better non-HDL cholesterol profiles than American males but in Indonesia this gender difference is inverted.

Controlling for age, body composition, health behaviors, and socioeconomic factors, we are unable to explain the between-country differences in HDL cholesterol, though for non-HDL risk measures that account for medication use, we are able to substantially attenuate between-country disparities, suggesting an important role for these factors in between-country disparities. Overall, the findings suggest different production functions for HDL and non-HDL cholesterol as components of cardiovascular risk, and underscore important questions about the production of cardiovascular risk with respect to gender, age and body composition.

Adverse levels of HDL cholesterol among Indonesians remain largely unexplained, suggesting that poor HDL among Indonesians is potentially a function of unmeasured genetics, behavior, and environment. HDL levels among Indonesians are worse at every BMI and age level, and the shapes of the production function with age and BMI are likely different relative to the patterns for Americans. This suggests a role for factors not included in the models that have an important impact on HDL cholesterol, that differ substantially across the two populations and that also differ by gender within Indonesia. Genetic factors and early life exposures are unlikely to fully explain these results because they are shared by males and females in Indonesia. Gender-specific differences in, for example, background inflammation may be implicated.

For non-HDL cholesterol, when accounting for the potential role of lipid-lowering drugs like statins, we are able to explain much of the cross-country gaps, suggesting potentially more similarity in the production of non-HDL cholesterol than blood non-HDL levels indicate. To some extent, this may also explain steeper gradients of BMI with non-HDL cholesterol in Indonesia compared with the U.S. However, the inverted gender gaps in Indonesia and the U.S. remained unexplained. Leveraging detailed household data, we are able to reconcile this inverted pattern as attributable in large part to unmeasured household factors.

Whereas relationships between gender, age, body composition and blood lipids in Indonesia are distinctive and unique, the gradients in education and socioeconomic status are broadly similar between countries. While gender differences in socioeconomic gradients have been previously documented, they do not appear to be a core driver of outcomes differences across countries in this study (Kavanagh et al., 2010, Murasko, 2008).

Several important epidemiological and clinical considerations emerge from this work and make the Indonesian context distinctive. While other countries in Asia have been documented to have lower HDL cholesterol levels than those in the U.S., those countries also have lower non-HDL cholesterol levels, so the net effect on cardiovascular disease risk is unclear (Patel et al., 2006). In contrast, our data from Indonesia shows both lower HDL cholesterol and higher total cholesterol.

Further, while medication for lowering non-HDL cholesterol is commonly used in the U.S., it is nearly nonexistent in Indonesia. As a result, some of the results from the outcome that includes either high non-HDL cholesterol or medication use do not parallel the results from direct measures of non-HDL cholesterol. While accounting for the role of these medications helps reconcile some of the potential differences in the biology of non-HDL cholesterol across contexts, the high levels of risk in Indonesia without medication reinforce the need for additional access to cholesterol-controlling medications in Indonesia.

To our knowledge, this study is unique in its investigation of the role of age, body composition, gender, and socioeconomic status in explaining the disparities seen between a low-resource setting and the U.S. The results contribute to a growing literature documenting adverse HDL and non-HDL cholesterol levels in South Asians and Southeast Asians living in Asia and the U.S. (Frank et al., 2014, Hussain et al., 2016, Kolluri et al., 2009). Previous literature has suggested that children in South Asia have higher BMI-adjusted blood pressure levels than children in the U.S. (Jafar Tazeen H. et al. 2005). In our study of Southeast Asian adults, adjusting for a rich array of factors, we find evidence suggesting higher-than-expected cardiovascular disease risk with respect to HDL, but we are able to explain the majority of differences in non-HDL by accounting for medication use. An improved understanding of the etiology of these disparate patterns may provide important information on addressing high cholesterol risks in Indonesia, as well as the role of anthropometric and socioeconomic factors around the world.

## Ethics statement

The study protocol for STAR was approved by the Duke University Campus Institutional Review Board and SurveyMeter, Indonesia, Institutional Review Board. Each STAR participant provided written informed consent. NHANES data are collected by the Centers for Disease Control; public use NHANES data files are used in this research.

## CRediT authorship contribution statement

**Ralph Lawton:** Conceptualization, Data curation, Formal analysis, Investigation, Methodology, Validation, Visualization, Writing – original draft, Writing – review & editing. **Elizabeth Frankenberg:** Conceptualization, Data curation, Formal analysis, Funding acquisition, Investigation, Methodology, Project administration, Supervision, Validation, Visualization, Writing – original draft, Writing – review & editing. **Teresa Seeman:** Conceptualization, Investigation, Methodology, Writing – review & editing. **Arun Karlamangla:** Conceptualization, Investigation, Methodology, Resources, Validation, Writing – review & editing. **Cecep Sumantri:** Investigation, Methodology, Project administration, Resources, Validation, Writing – review & editing. **Duncan Thomas:** Conceptualization, Data curation, Formal analysis, Funding acquisition, Investigation, Methodology, Project administration, Supervision, Validation, Visualization, Writing – original draft, Writing – review & editing.

## Figures and Tables

**Table 1:** Regression Results Relating POCT and Gold-Standard Laboratory Paired Values of HDL and non-HDL Cholesterol under different field conditions.

	POCT Measured Levels					
	HDL Cholesterol			Non-HDL Cholesterol		
Gold-Standard Lab	Ideal	Field-Uncontrolled	Field-Climate Controlled	Ideal	Field-Uncontrolled	Field-Climate Controlled
Slope coefficient	1.23	1.12	1.16	0.81	0.88	0.95
	[1.11–1.34]	[0.97–1.28]	[1.04–1.27]	[0.74–0.88]	[0.76–0.99]	[0.84–1.07]
Constant	0.33	-6.16	1.72	-2.46	0.11	7.86
	[-0.76–1.42]	[-8.05 - -4.27]	[0.54–2.91]	[-5.14–0.21]	[-3.83–4.05]	[3.83–11.89]
Observations	107	50	130	107	50	54
R-squared	0.81	0.82	0.75	0.83	0.84	0.84

Notes: Regressions compare findings with paired-sample assessments drawn where blood draws and POCT measures using the CardioChek were conducted under three conditions: ideal, uncontrolled field, and climate-controlled field conditions. OLS regressions predict the POCT measures from the field with the gold-standard laboratory-assessments, and 95% heteroscedasticity-consistent confidence intervals are reported below coefficients.

**Table 2:** Proportion clinically-relevant HDL and non-HDL POCT measures correctly classified across conditions under which measures were collected.

	Gold-Standard Laboratory Classifications				
A. Ideal Conditions					
	Normal	Low HDL		Normal	High Non-HDL
Normal	**0.48**	0.07	Normal	**0.76**	0.10
Low HDL	0.07	**0.37**	High Non-HDL	0.02	**0.12**
B. Uncontrolled Field Conditions					
	Normal	Low HDL		Normal	High Non-HDL
Normal	**0.46**	0.00	Normal	**0.78**	0.08
Low HDL	0.36	**0.18**	High Non-HDL	0.04	**0.10**
C. Controlled field conditions					
	Normal	Low HDL		Normal	High Non-HDL
Normal	**0.61**	0.08	Normal	**0.83**	0.00
Low HDL	0.07	**0.25**	High Non-HDL	0.09	**0.07**

Notes: Low HDL Cholesterol is <40 mg/dL and High Non-HDL Cholesterol is >190 mg/dL

**Table 3:** Lipid Profiles in other Asian populations. Means (standard errors) of respondents age 20 and older.

	**HDL Cholesterol**						**Non-HDL Cholesterol**					
	Level			%<40			Level			%>190 | medication		
	Males	Females	M-F Gap	Males	Females	M-F Gap	Males	Females	M-F Gap	Males	Females	M-F Gap
OTHER ASIAN POPULATIONS												
1. China (CHNS)												
Unadjusted	53.4	56.9	-3.5	17.7	9.3	8.5	134.3	134.5	-0.2	7.9	8.8	-0.9
	(0.3)	(0.2)	(0.4)	(0.7)	(0.5)	(0.8)	(0.7)	(0.6)	(0.9)	(0.5)	(0.5)	(0.7)
Age adjusted	53.1	57.5	-4.4	17.9	8.6	9.3	131.6	131.4	0.2	7.1	8.3	-1.2
	(0.3)	(0.3)	(0.4)	(0.8)	(0.5)	(0.9)	(0.8)	(0.7)	(1.1)	(0.5)	(0.5)	(0.7)
Sample sizes	3,077	3,653		3,077	3,653		3,077	3,653		3,077	3,653	
2. Korea (KNHANES)												
Unadjusted	42.3	47.4	-5.2	49.3	26.1	23.2	143.6	134.3	9.3	8.7	6.6	2.1
	(0.4)	(0.4)	(0.5)	(1.8)	(1.6)	(2.4)	(1.2)	(1.2)	(1.7)	(1.0)	(0.8)	(1.3)
Age adjusted	42.6	46.6	-4.0	48.3	30.9	17.5	141.1	141.2	-0.1	7.2	8.9	-1.8
	(0.2)	(0.2)	(0.3)	(1.1)	(0.9)	(1.5)	(0.8)	(0.7)	(1.0)	(0.6)	(0.6)	(0.8)
Sample sizes	2,315	3,124		2,315	3,124		2,314	3,124		2,314	3,124	

3. Indonesia (IFLS)												
Unadjusted	34.4	43.8	-9.5	70.7	44.2	26.6	143.6	150.9	-7.3	15.4	19.5	-4.0
	(0.3)	(0.3)	(0.4)	(0.9)	(0.9)	(1.2)	(0.9)	(0.9)	(1.2)	(0.7)	(0.7)	(1.0)
Age adjusted	34.3	44.0	-9.7	73.7	38.2	35.5	140.1	155.5	-15.4	12.5	19.1	-6.6
	(0.5)	(0.7)	(0.8)	(2.2)	(3.7)	(4.3)	(2.5)	(3.1)	(4.0)	(1.1)	(2.2)	(2.4)
Sample Sizes	3,610	4,258		3,610	4,258		3,608	4,256		3,608	4,256	

Notes: Coefficients for proportion over cutoffs multiplied by 100 to represent percentages. Age-adjustments reflect re-weighting of non-US samples in 5-year age groups to match the proportions in the NHANES population. Heteroscedasticity-consistent robust standard errors in parentheses.

## Data Availability

Data will be made available on request.

## Footnotes

1 Gender is used to reflect differences between males and females that may be biological and/or related behaviors, access to resources and social contexts.

2 In the 2015 wave of IFLS, cholesterol was measured in the home for a sample of respondents who are representative of about 80% of the Indonesians at the time of the 1993 baseline. Both HDL and total cholesterol measures were low relative to estimates from clinical samples. One arm of our validation study for the STAR sample replicated the IFLS protocol but also collected a paired sample from each validation study respondent that was assayed in a clinical laboratory. Comparing the two measures, we established that the in-home measures collected in IFLS were significantly and substantially biased downwards. Our analyses of the IFLS data, reported in panel 3 of Appendix Table 3, indicate that this is driven by low levels of measured HDL cholesterol which is also reflected in total cholesterol. However, on average, non-HDL cholesterol levels match our data from Aceh more closely and the underlying gender epidemiology is very similar in the two studies, indicating that our results for Aceh likely generalize to the broader Indonesian population. In STAR, sample collection and measurement of total and HDL cholesterol were conducted in our mobile mini-lab with temperature and humidity carefully controlled. IFLS sample collection and measurements were conducted in the home. The differences suggest that collection and measurement conditions affected HDL cholesterol but non-HDL cholesterol appears to have been less affected.

3 Following the Third Report of the National Cholesterol Education Program (NCEP) Expert Panel on Detection, Evaluation, and Treatment of High Blood Cholesterol in Adults (Adult Treatment Panel III)’s recommendations for non-fasting measures, we defined low non-fasting HDL cholesterol to be HDL<40 mg/dL and high non-HDL cholesterol to be non-HDL> 190 mg/dL (National Cholesterol Education Program (NCEP) Expert Panel (Adult Treatment Panel III) 2002).

4 Regression models that predict non-HDL cholesterol include a control for whether the respondent is on cholesterol-reducing medication.

5 NHANES measures body fat and body fat allocation with dual x-ray absorptiometry (DEXA) scans but STAR uses bioelectrical impedance. The two measures are not comparable and are not included in the models.

6 We have investigated the importance of clustered standard errors at the primary sampling unit in the NHANES and at the community of residence at the baseline in STAR. None of our substantive conclusions is changed: standard error estimates are very similar to those reported in our tables, suggesting a relatively small role for within-cluster correlated errors in these analyses.

7 The estimates are LOWESS (Cleveland, 1979) with bandwidths spanning 25% of the data.

8 Taken together, BMI and waist circumference are jointly significant in all of the gender- and country-specific models of cholesterol levels (p-values< 0.01).

9 Replacing lnPCE with lnPCY in the Indonesian sample does not affect this conclusion. Adjusting for purchasing power parity only affects the intercept in the models.

10 We have experimented with including a potential measure of relative bargaining power within a household. Specifically, being better educated than one’s spouse should not affect cholesterol directly through the health production function or budget constraint under the assumptions of a unitary model of decision-making; it may, however, affect leverage over resource allocations within the household. We find that a male who is better educated than his spouse has a higher HDL level and is less likely to present with low HDL; the latter difference is large in magnitude (12.3 percentage points) and statistically significant. Non-HDL is not similarly affected for males. There is no evidence that any of the cholesterol indicators varies depending on whether a female is better educated than her spouse. We conclude there is some evidence that within household bargaining power may be implicated in explaining the gender difference in HDL cholesterol, especially among those with levels of HDL below the cut-off that indicates elevated cardiovascular risk.
